# Effect of ultrasound-guided transversus abdominis plane block with rectus sheath block on patients undergoing laparoscopy-assisted radical resection of rectal cancer: a randomized, double-blind, placebo-controlled trial

**DOI:** 10.1186/s12871-021-01295-9

**Published:** 2021-03-24

**Authors:** Min Liang, Xia Xv, Chunguang Ren, Yongxing Yao, Xiujuan Gao

**Affiliations:** 1 Department of Anesthesiology, Liaocheng People’s Hospital, Liaocheng, Shandong People’s Republic of China; 2 Department of Anesthesiology, First Affiliated Hospital, Zhejiang University School of Medicine, Hangzhou, Zhejiang People’s Republic of China

**Keywords:** Ultrasound, Transversus abdominis plane, Rectus sheath, Sufentanil, Rectal cancer

## Abstract

**Background:**

Many patients complain of pain following laparoscopic surgery. Clinicians have used ultrasound-guided posterior transversus abdominis plane block (TAPB) and rectus sheath block (RSB) for multimodal analgesia after surgery. We investigated the analgesic effects of US-guided posterior TAPB with RSB on postoperative pain following laparoscopy-assisted radical resection of early-stage rectal cancer.

**Methods:**

Seventy-eight adults scheduled for laparoscopy-assisted radical resection of rectal cancer were enrolled in this double-blind placebo-controlled trial. Patients were randomized into 3 groups: the TR Group underwent US-guided bilateral posterior TAPB (40 mL 0.33% ropivacaine) with RSB (20 mL 0.33% ropivacaine); the T Group underwent US-guided bilateral posterior TAPB alone; and the Control Group received saline alone. All patients also had access to patient-controlled intravenous analgesia (PCIA) with sufentanil. The primary outcome was postoperative sufentanil consumption at 0–24, 24–48, and 48–72 h. The secondary outcomes were postoperative pain intensity and functional activity score at rest and while coughing for the same three time periods, intraoperative medication dosage, use of rescue analgesia, recovery parameters, and adverse effects.

**Results:**

The three groups had no significant differences in baseline demographic and perioperative data, use of intraoperative medications, recovery parameters, and adverse effects. The TR group had significantly lower postoperative use of PCIA and rescue analgesic than in the other two groups (*P* < 0.05), but the Control Group and T Group had no significant differences in these outcomes.

**Conclusions:**

Postoperative US-guided posterior TAPB with RSB reduced postoperative opioid use in patients following laparoscopy-assisted radical resection of rectal cancer.

**Trial registration:**

The trial was registered with chictr.org (ChiCTR2000029326) on January 25, 2020.

## Background

Colorectal cancer is one of the most common tumors and is a leading cause of cancer-related deaths worldwide [1]. Because of the increasing incidence of rectal cancer and the improved rates of recovery after surgery, laparoscopy-assisted radical resection of early-stage rectal cancer has become more common [2]. Although laparoscopy reduces the size of the operative incision, many patients complain of postoperative pain. Opioid analgesia is traditionally provided following a laparoscopic abdominal operation [3]. However, adverse effects from opioid analgesia (postoperative nausea and vomiting [PONV]), may increase the duration of the hospital stay and reduce patient satisfaction [4].

There has been an increasing use of ultrasound (US) technologies, and US-guided peripheral nerve block has become a fundamental part of postoperative multimodal analgesia. US-guided posterior transversus abdominis plane block (TAPB) and US-guided rectus sheath block (RSB) have been used during abdominal surgeries, and previous studies indicated that they provide potent analgesic effects [5–7]. Currently, there is limited evidence in the literature to support the use of US-guided posterior TAPB combined with RSB.

The aim of this study is to evaluate the efficacy of US-guided posterior TAPB with or without RSB in postoperative pain management for patients following laparoscopy-assisted radical resection of rectal cancer.

## Methods

### Patients

This randomized, double-blinded, placebo-controlled trial was performed following approval of the ethics committee of the Liaocheng People’s Hospital. The trial was registered with chictr.org before enrollment of the first participant (Trial registration: ChiCTR, ChiCTR2000029326. Registered 25 January 2020, http://www.chictr.org.cn), and written informed consent was obtained from all patients prior to enrollment. All findings are reported in accordance with the Consolidated Standards of Reporting Trials (CONSORT) guidelines.

All enrolled patients were scheduled to undergo laparoscopic radical resection (Dixon operation) following diagnosis of clinical stage I or II rectal cancer at the Liaocheng People’s Hospital between January 2020 and June 2020. The inclusion criteria were age between 35 and 70 years, ability to understand and use the pain assessment method, and physical status of I to III according to the American Society of Anesthesiologists (ASA). The exclusion criteria were history of allergy to local anesthetics, history of opioid abuse, history of treatment for another cancer, refusal to use patient-controlled analgesia (PCA), need for resection of another organ(s) in addition to the rectum, history of previous abdominal surgery, and preoperative intestinal obstruction requiring emergency surgery.

### Randomization and blinding

Two days before surgery, random numbers that were generated by SPSS were used to assign equal numbers of eligible participants to one of the three groups. The TR Group received US-guided bilateral posterior TAPB with 40 mL of 0.33% ropivacaine and RSB with 20 mL of 0.33% ropivacaine; the T Group received US-guided bilateral posterior TAPB with 40 mL of 0.33% ropivacaine and RSB with 20 mL of 0.9% normal saline; the Control Group received US-guided bilateral posterior TAPB with 40 mL of 0.9% normal saline and RSB with 20 mL of 0.9% normal saline. Patients in all groups also received postoperative patient controlled intravenous analgesia (PCIA) (see below for details). Randomized results were kept in a sealed envelope and relayed to an independent nurse anesthetist who prepared the drug or placebo on the morning of the operation. The remainder of the clinicians, the main anesthesiologist, and the anesthesiologist who administered the TAPB were all blinded to the group allocations.

### General anesthesia and monitoring

All patients underwent regular fasting for 8 to 12 h without any preoperative medication. Before the induction of anesthesia, intravenous (IV) fluids were administered at a rate of 10 mL/kg/h, and the heart rate, continuous invasive arterial blood pressure, pulse oxygen saturation, bispectral index (BIS), and nasopharyngeal temperature were continuously monitored using a multifunction monitor (Philips IntelliVue MP50, Boeblingen, Germany). For all patients, anesthesia was induced with IV propofol (2.0 mg/kg), fentanyl (3 μg/kg), and cisatracurium (0.15 mg/kg), according to standard general anesthesia guidelines set by the institute. Tracheal intubation was performed after muscle relaxation. Anesthesia was maintained using inhalational sevoflurane and remifentanil. To maintain a BIS value of 40 to 60 and a mean arterial pressure within 20% of the baseline value, sevoflurane and remifentanil were continuously adjusted. Ephedrine was administrated if the intraoperative blood pressure was more than 20% below the baseline, and atropine was administered if the heart rate was lower than 50 bpm. IV cisatracurium (0.03 mg/kg) was added hourly until the end of the operation.

### Surgery

**Fig. 1:**
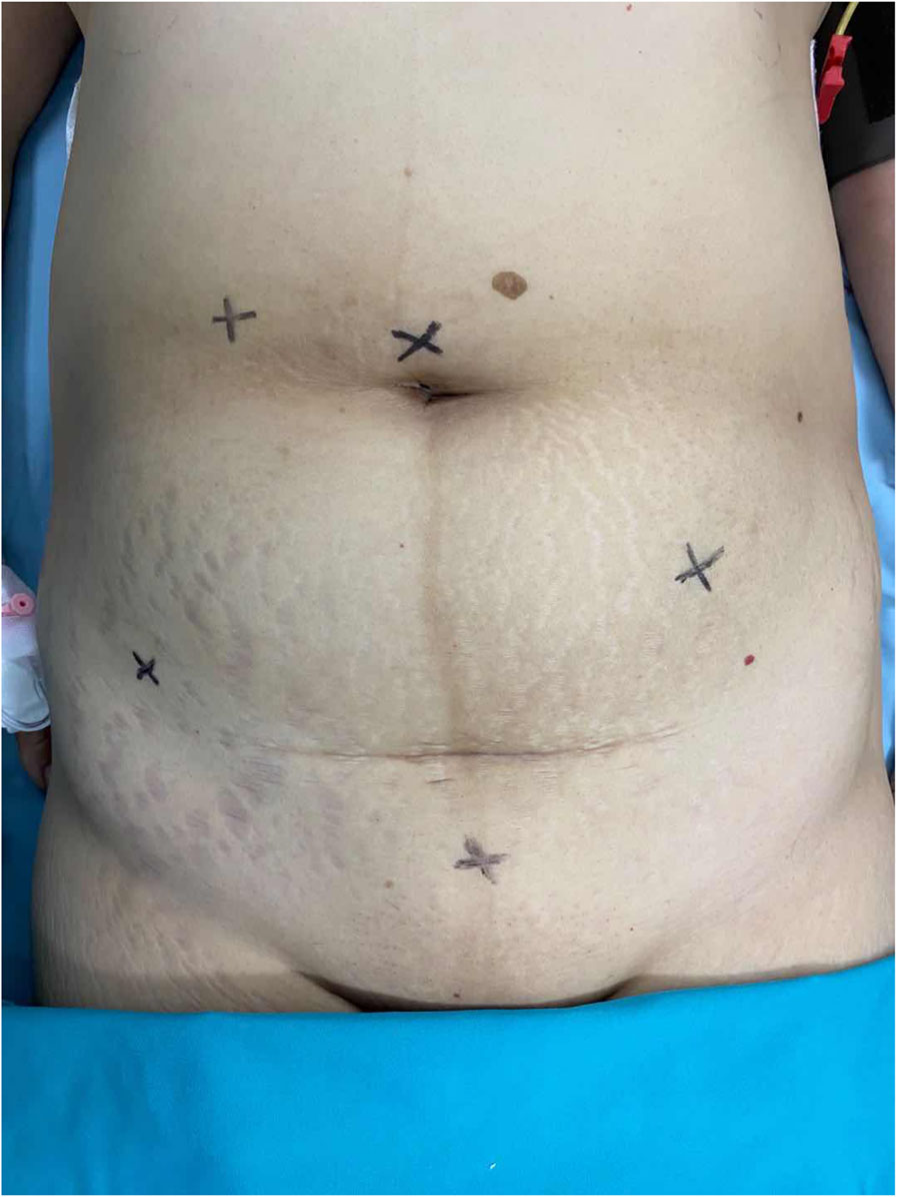
Laparoscopic incision and trocar insertion using the five hole method

Laparoscopic incision and trocar insertion were performed by adopting the five hole method. The five incisions were: *(i)* on the supraumbilical site; *(ii)* 2 finger widths inside the right anterior superior iliac spine; *(iii)* 3 to 4 cm above the cross point of the right clavicular midline and the umbilicus intersection; *(iv)* at the mid-point of the line between the left anterior superior iliac spine and umbilicus; and *(v)* 2 finger widths above the symphysis pubis, which could be expanded to 5 or 6 cm for specimen removal (Fig. 1). The pneumoperitoneum was established with carbon dioxide, and pressure was 12 to 14 mmHg (1.36 to 1.58 kpa). The operation was performed according to the specifications and principles of laparoscopic and radical resection of rectal cancer (i.e. total mesorectal resection (TEM), thorough lymphadenectomy, and tumor eradication). The Dixon operation was used based on tumor location.

### US-guided TAPB and RSB

**Fig. 2:**
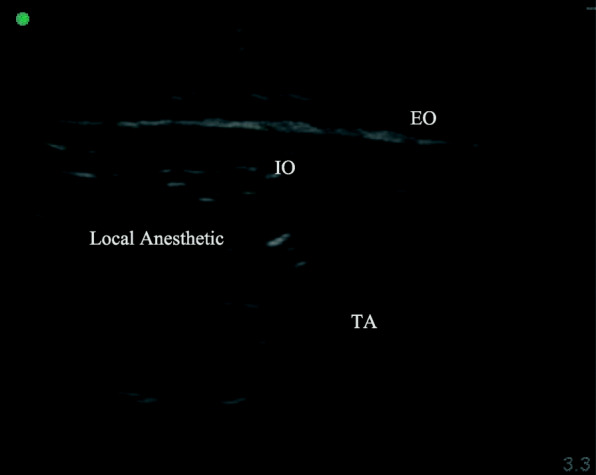
TAP block

**Fig. 3:**
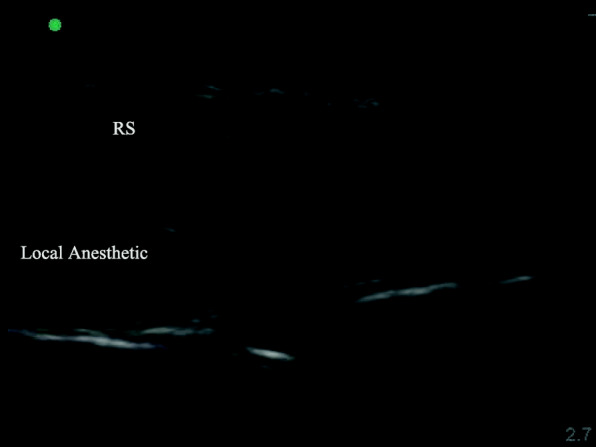
RS block

After removal of the tracheal tube, TAPB was performed immediately by a qualified anesthesiologist using US guidance (SonoSite S-Nerve Ultrasound System) and a broadband (4 to 13 MHz) linear array ultrasound probe. For the posterior approach, the probe was placed transversely in the midaxillary line between the iliac crest and the costal margin [8]. Then, the probe was moved outward and the needle was inserted when the TAP was identified. When the tip of the needle was in the TAP, 2 mL of normal saline was injected to adjust its position. Then, 40 mL of 0.33% ropivacaine was administered to the TR Group and T Group, and 40 mL of 0.9% normal saline was administered to the Control Group (Fig. 2). Next, RSB was performed on both sides of the linea alba under US-guidance [7]. For the RSB, the probe was placed transversely on the rectus abdominis and the needle was inserted using US guidance until the tip was in the plane between the rectus abdominis and the posterior sheath of the rectus abdominis [9]. Patients in the TR Group received 20 mL of 0.33% ropivacaine, and patients in the T Group and Control Group received 20 mL of 0.9% normal saline. The procedure was divided into 3 or 4 injection sites on the left and right sides of the surgical site to prevent the rectus abdominis tendon from blocking the spread of the drug (Fig. 3).

### Postoperative care

Sufentanil was used for PCIA, which was initiated in the post-anesthesia care unit (PACU). The PCIA regimen consisted of 300 mL of 0.8 μg/mL sufentanil, with a bolus dose of 2 mL, a lockout time of 5 min with no background infusion, and a 4-h maximum limit of 30 mL (24 μg). The aim of PCIA was to control pain intensity based on a numerical rating scale (NRS) at rest of 4 or less. The NRS is an objective pain intensity assessment tool that has a scale of 0 to 10, in which 0 indicates no pain and 10 indicates the worst pain possible. Any patient whose NRS value at rest was above 4 was given a loading dose of 4 mL (3.2 μg) sufentanil, and had a 4 h maximum limit of 40 mL. For patients with insufficient analgesia or sufentanil intolerance, additional rescue analgesia was given (30 mg IV ketorolac).

### Data collection

Sufentanil-based PCIA was used in all groups with the same regimen for 72 h after surgery. The cumulative PCA usage during three time period after the operation (0 to 24 h, 24 to 48 h, and 48 to 72 h) was the primary outcome, and was recorded by a blinded member of the acute pain service (APS) team. The secondary outcomes were postoperative pain intensity on the NRS and functional activity score (FAS) at rest and during coughing after 24, 48, and 72 h [10]. The FAS is a subjective pain intensity assessment tool that uses grades A, B, and C. Grade A indicates that functional activity is not limited by pain; grade B indicates that functional activity is moderately limited because of pain; and grade C indicates that functional activity is severely limited because of pain. Use of intraoperative medications and rescue analgesia were recorded. In addition, the time to first flatus, defecation, oral intake, and discharge were recorded. All adverse effects possibly due to sufentanil, such as nausea, vomiting, pruritus, respiratory depression, and dizziness, were recorded.

### Statistical analysis

The sample size was based on the initial pilot data, in which the means ± standard deviations of sufentanil use during the 24 h period after surgery were recorded. (Control Group: 84 ± 47 μg, T Group: 80 ± 37 μg, TR Group: 41 ± 16 μg). Based on a power of 95% and a significance level of 5%, 20 patients per group were necessary. Assuming a 30% dropout rate, a minimum of 26 patients per group were enrolled.

Statistical analysis was performed using SPSS 16.0 (SPSS Inc. Chicago, IL). The Kolmogorov-Smirnov test was used to assess the distributions of variables, and homogeneity of variance was determined using Levene’s test. Data with normal distributions are presented as means and standard deviations and data with non-normal distributions are presented as medians and interquartile ranges. Categorical data are presented as number (n) and percentage (%). ANOVA tests were used to compare data with normal distributions. Non-parametric tests were used to compare data with non-normal distributions; the Kruskal-Wallis H method was used for overall comparisons, and the Mann-Whitney U method was used to compare differences between groups. Categorical variables were analyzed using the χ^2^ test or Fisher’s exact test. *P* values below .05 were considered significant.

## Results

**Fig. 4:**
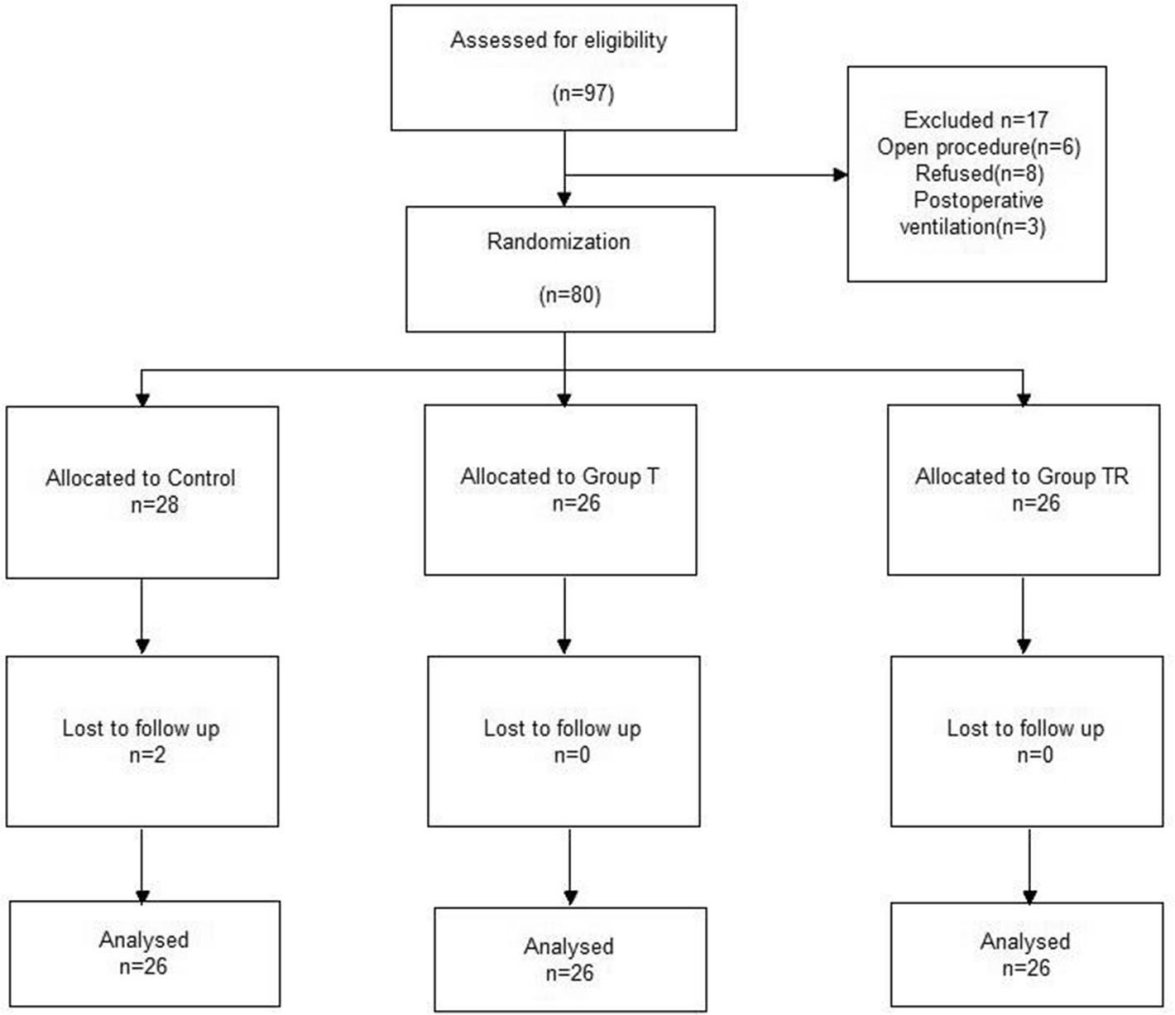
Disposition of eligible patients with stage I/II rectal cancer

Our assessments indicated that 97 patients were eligible for enrollment (Fig. 4) Seventeen patients were excluded and 2 patients were lost to follow-up. We therefore analyzed data of the remaining 78 patients. The 3 groups had no significant differences in demographic parameters, operation conditions, and fluid and anesthetic administration (*P* > 0.05, Table 1). In addition, none of the patients required a change of analgesic.

**Table 1:** Demographic and perioperative characteristics of the three groups

Variable	Group C (*n* = 26)	Group T (*n* = 26)	Group TR (*n* = 26)	*P*
Age (years)	60.6 ± 8.2	61.5 ± 8.2	61.5 ± 8.1	0.899
Male/Female (n)	13/13	16/10	18/8	0.362
BMI (kg/m^2^)	23.6 ± 2.2	23.4 ± 2.0	23.9 ± 2.6	0.697
ASA I/II/III (n)	1/21/4	1/18/7	0/19/7	0.691
Clinical Stage I/II (n)	14/12	10/16	13/13	0.513
Blood loss (mL)	108.0 ± 65.4	131.9 ± 30.1	131.5 ± 26.6	0.092
Urine (mL)	398.0 ± 226.5	301.9 ± 197.7	380.8 ± 183.9	0.199
Surgery duration (min)	174.6 ± 50.2	195.2 ± 46.0	181.7 ± 42.7	0.275
Fluid infusion (mL)	1465.4 ± 386.7	1511.5 ± 436.3	1536.5 ± 437.6	0.833
Propofol (mg)	120.0 ± 21.9	119.6 ± 17.5	128.3 ± 18.3	0.197
Fentanyl (mg)	0.27 ± 0.08	0.29 ± 0.08	0.29 ± 0.09	0.765
Cisatracurium (mg)	20.1 ± 3.7	21.0 ± 3.2	19.8 ± 3.3	0.412
Remifentanil (mg)	0.38 ± 0.13	0.46 ± 0.12	0.42 ± 0.10	0.065
Sevoflurane (%)	2.47 ± 0.37	2.44 ± 0.37	2.32 ± 0.40	0.336
Atropine (mg)	2(7.7%)	3(11.5%)	4(15.4%)	0.690
Ephedrine (mg)	5(19.2%)	3(11.5%)	2(7.7%)	0.448

Variables are presented as mean ± SD or number of patients (n)

Data were analyzed using a one-way analysis of variance or chi-square test. *ASA* American Society of Anesthesiology, *BMI* body mass index

**Fig. 5:**
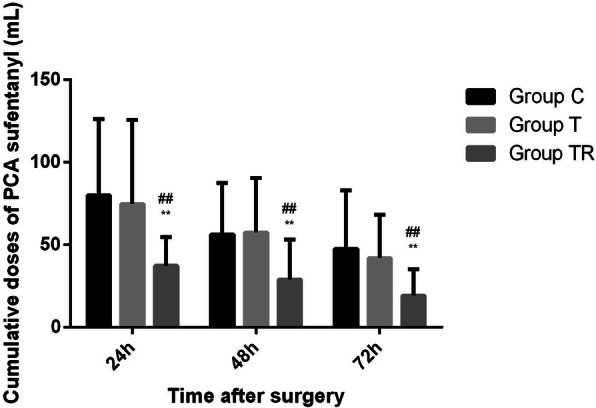
Postoperative sufentanil use in the three groups. **P < .01 vs. Control Group, ##*P* < .01 versus. T Group

The TR Group used significantly less postoperative sufentanil than the Control Group and Group T at 0 to 24 h (37.5 ± 17.38 μg vs. 80.0 ± 46.13 μg vs. 74.8 ± 51.10, *P* < 0.01), 24 to 48 h (29.0 ± 24.28 μg vs. 56.3 ± 31.31 μg vs. 57.6 ± 32.86, *P* < 0.01), and 48 to 72 h (19.4 ± 15.84 μg vs. 47.6 ± 35.41 μg vs. 42.0 ± 26.24, *P* < 0.01) (Fig. 5). The Control Group and T Group had no significant difference in use of postoperative sufentanil (*P* > 0.05, Fig. 5).

**Fig. 6:**
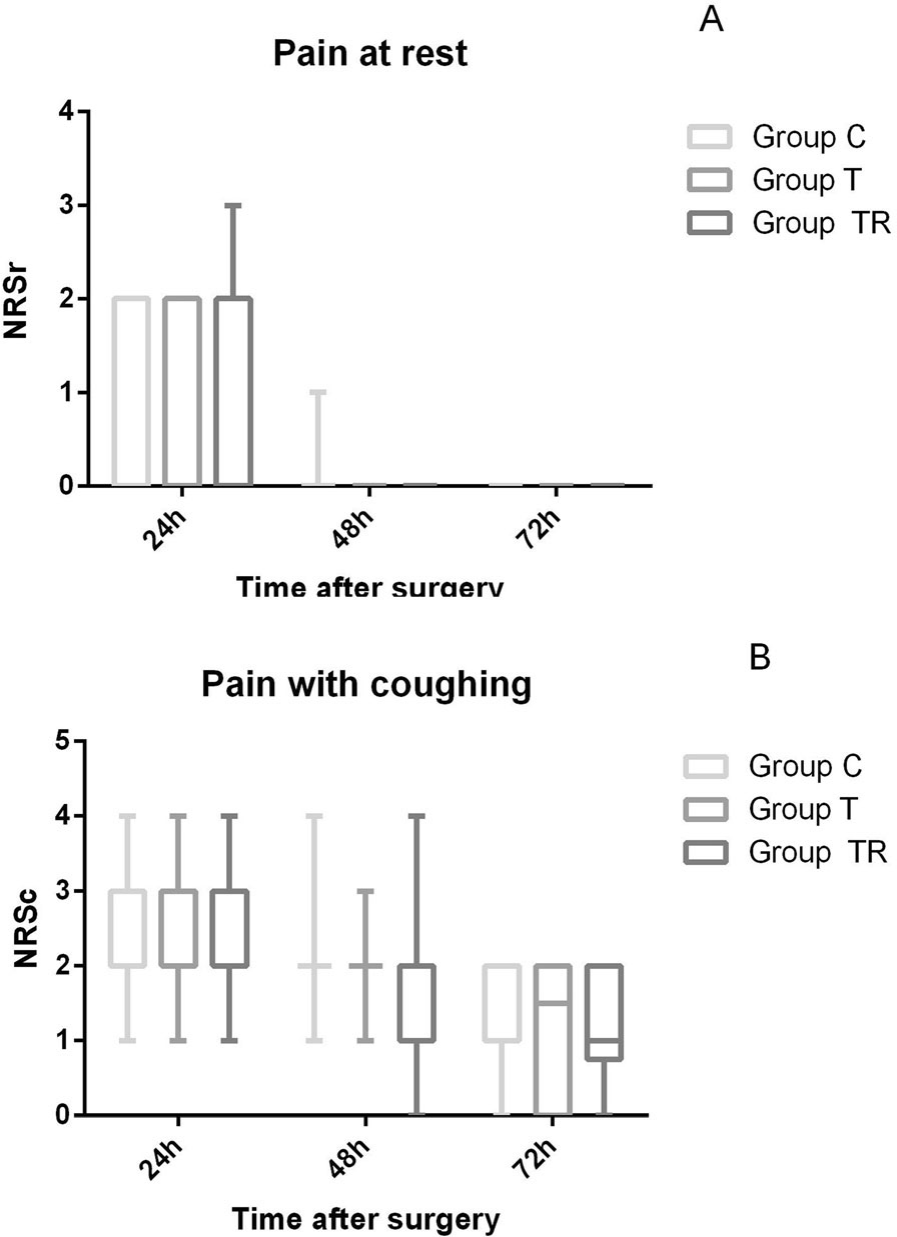
**a** Postoperative pain scores at rest in the three groups (NRS = numerical rating scale). **b** Postoperative pain scores with coughing in the three groups

The postoperative NRS pain scores at rest and during coughing were low in all groups at 24 h, 48 h, and 72 h, and there were no significant differences among the three groups (*P* > 0.05, Fig. 6). The postoperative FASs were also favorable in all groups at 24 h, 48 h, and 72 h, and there were no significant differences (*P* > 0.05, Table 2).

**Table 2:** Postoperative functional activity scores in the three groups (n)

	Variable	Group C (*n* = 26) C/B/A	Group T (*n* = 26) C/B/A	Group TR (*n* = 26) C/B/A	*P*
FAS	24 h	0/4/22	0/2/24	0/3/23	0.686
	48 h	0/2/24	0/1/25	0/0/26	0.353
	72 h	0/1/25	0/0/26	0/0/26	0.363

Variables are presented as number of patients (n)

Data were analyzed using the chi-square test

Functional Activity Score (FAS): A, not limited (functional activity not limited because of pain); B, mild to moderate limitation (functional activity mildly to moderately limited because of pain); C, severely restricted (functional activity severely limited because of pain)

The TR Group required less rescue analgesia than the Control Group and the T Group (*P* < 0.05, Table 3), but there were no significant differences between the Control Group and the T Group (*P* > 0.05, Table 3). The three groups had no significant differences in any of the measured recovery parameters (*P* > 0.05, Table 4) and no differences in the incidences of sufentanil-associated adverse effects (*P* > 0.05, Table 5). None of the patients experienced respiratory depression (*P* > 0.05, Table 5).

**Table 3:** Use of rescue analgesia in the three groups

Variable	Group C (*n* = 26)	Group T (*n* = 26)	Group TR (*n* = 26)	*P*
Rescue analgesic	11(42.3%)	8(30.8%)	2(7.7%)*	0.016

Variables are presented as number of patients n (%)

Data were analyzed using the chi-square test

* *p* < 0.05 versus Group T or Group C

**Table 4:** Recovery parameters in the three groups

Variable	Group C (*n* = 26)	Group T (*n* = 26)	Group TR (*n* = 26)	*P*
Time to first flatus (h)	63.4 ± 8.71	64.6 ± 7.80	65.6 ± 6.35	0.604
Time to first solid food (h)	81.5 ± 7.64	82.9 ± 6.80	84.5 ± 6.83	0.283
Time to first feces (h)	132.3 ± 13.16	133.1 ± 14.73	126.5 ± 13.82	0.192
Postoperative ileus	1 (3.8%)	1 (3.8%)	2 (7.7%)	0.768
Duration of postoperative hospital stay (days)	6.5 ± 1.01	6.7 ± 0.96	6.8 ± 1.08	0.590

Variables are presented as mean ± SD or number of patients n (%)

Data were analyzed using a one-way analysis of variance or the chi-square test

**Table 5:** Adverse effects in the three groups

Variable	Group C (*n* = 26)	Group T (*n* = 26)	Group TR (*n* = 26)	*P*
Nausea	5(19.2%)	4(15.4%)	6(23.1%)	0.781
Vomiting	2(7.7%)	1(3.8%)	2(7.7%)	0.808
Puritus	2(7.7%)	1(3.8%)	0(0%)	0.353
Respiratory depression	0	0	0	1.000
Dizziness	3(11.5%)	1(3.8%)	1(3.8%)	0.425

Variables are presented as number of patients n (%)

Data were analyzed using the chi-square test

## Discussion

There are two main findings in this research. First, patients who received laparoscopy-assisted radical resection of early-stage rectal cancer had significantly reduced use of postoperative PCIA and rescue analgesia when they received US-guided TAPB with RSB. Second, there were no significant differences in the use of postoperative PCIA for groups who received US-guided posterior TAPB with RSB and TAPB alone.

A laparoscopic approach for colorectal surgery has many benefits for patients, and should now be considered as a standard treatment [11–13]. Nevertheless, postoperative pain is still the main factor affecting the recovery of these patients [14], and adequate postoperative analgesia remains essential [15, 16]. Multimodal analgesia, including US-guided peripheral nerve block, has proven safety and effectiveness for pain management [17–19]. For example, a retrospective study of 43 patients undergoing laparoscopic colectomy showed that the combination of US-guided TAP and RSB significantly reduced the use of continuous intravenous fentanyl after surgery [20]. Another prospective study of 60 patients showed that TAP (20 mL of 0.375% ropivacaine) significantly reduced the use of fentanyl by patients undergoing hand-assisted laparoscopic colon surgery, and also reduced the recovery time of bowel function and the duration of hospitalization [21]. Khaled Yassen et al. observed 55 patients with cirrhosis who received liver surgery and found that repeated US-guided TAPB and RSB (0.2 mL/kg of 0.25% bupivacaine, every 8 h for 48 h) significantly reduced the use of opioids after surgery [19]. Khaled Abdelsalam et al. observed 40 patients undergoing upper abdominal surgery (hepatectomy or Whipple procedure) and found that US-guided bilateral TAPB and RSB (20 mL of bupivacaine 0.25% for each) significantly reduced the use of opioids [22]. Another prospective study of 126 patients who received laparoscopic liver resection showed that US-guided bilateral TAP and RSB significantly reduced the patient-reported pain-VAS score, the dosage of ondansetron, and hospital stay [23]. Lili Xu et al. found that US-guided TAPB plus RSB prolonged the analgesia time and reduced postoperative pain in high-risk elderly patients who received emergency abdominal surgery [24]. Other studies of single-incision gynecological laparoscopic surgery reported that bilateral TAPB combined with RSB provided better results than TAPB alone [25, 26]. In addition, Tak Kyu Oh et al. examined patients undergoing laparoscopic colorectal cancer surgery and reported that TAP did not significantly reduce postoperative pain or opioid use [27]. The conclusions of these many studies are consistent with our findings.

Rafi et al. first described postoperative TAPB as a peripheral nerve block in 2001 [28]. Because of improvements in US technologies, anesthesiologist now commonly use US-guided TAPB for perioperative pain management [6, 18]. A review by Wu et al. concluded that TAP reduced the pain score at 6 h after surgery and the use of analgesics at 24 h after surgery [29]. Walter et al. found that preoperative TAP block in patients who received laparoscopic colorectal resection led to reduced opioid use, but had no effect on pain-VAS scores and median hospital stay [30]. These results are contrary to our findings.

There may be several reasons for these contradictory results. First, laparoscopy-assisted radical resection of rectal cancer requires two incisions above the umbilicus, and there may be variations among patients in the pain arising from these incisions. There is evidence that the cephalad dermatome levels achieved by posterior TAP is at T_10_, which is more suitable for analgesia of the incision below the umbilicus [8, 31]. This could explain our failure to observe a benefit from the TAP block. For upper abdominal incisions, US-guided RSB might be a better alternative for injecting local anesthetics into the posterior rectus sheath [32]. The ventral branch of the T_7_-T_12_ intercostal nerve can be blocked, thus anesthetizing the anterior wall of the abdomen from the xiphoid process to the pubic symphysis [33]. There is evidence that RSB blocks the anterior branch of T_9_-T_11_, and provides a better analgesic effect for incision around the umbilicus [34]. RSB could be performed alongside posterior TAPB to block higher dermatomes in the abdominal wall, up to T_6_ [22]. There is also evidence that TAPB reduces the use of fentanyl by 20%, and that RSB combined with TAPB reduces the use of fentanyl by more than 60% in patients with cirrhosis undergoing liver resection [19].

A second reason for these contradictory results may be that different approaches for TAP lead to different analgesic effects. There are two major approaches used for TAP: a lateral approach and the more conventional posterior approach. In the latter approach (which we used), the US probe is placed between the costal margin and the iliac crest at the axillary midline, and then scanned backward until the transverse abdominal muscle moved into the aponeurosis. The local anesthetic is then injected into the TAP near the aponeurosis, and a wider spread of local anesthetic could provide sufficient analgesia. Previous research demonstrated that the drug spreads to the paravertebral space and creates a paravertebral block that relieves visceral pain during lower abdominal surgery [5, 35].

A third possible reason for the contradictory results may be that there is a lack of uniform standards regarding the doses of local anesthetics used during US-guided TAPB.

We found no significant difference in the pain-VAS score or the FAS among the three groups, because all patients used PCIA and rescue analgesics when necessary to control the pain-VAS score below 4. There was also no significant difference in the measured recovery parameters and sufentanil-associated adverse effects among the three groups, but this may be due to the small sample size.

This study had some limitations that should be considered. First, we did not study the effect of the duration of analgesia provided by the peripheral nerve block. Previous studies showed that the analgesia duration from a single-shot TAPB lasts for 24 to 48 h, but it is possible that our use of two nerve blocks and the poor vascularization of the TAP and RS prolonged the duration of analgesia. Second, we did not perform blood concentration monitoring because previous studies showed that the blood concentration of ropivacaine did not exceed the toxic threshold of 2.2 μg/mL when 60 mL of ropivacaine 0.375% was used in US-guided TAP [36, 37]. Third, the study indicated that TAPB with RSB afforded better analgesic effect than TAPB or placebo, although we did not compare the analgesic effect of RSB and TAPB. Further research on this topic is necessary. Finally, this was a single center study, so our results cannot be generalized to other medical centers.

## Conclusions

In conclusion, we found that postoperative US-guided posterior TAPB with RSB significantly reduced postoperative opioid use by patients following laparoscopy-assisted radical resection of rectal cancer. Further studies are required to determine the duration of analgesia provided by this procedure.

## Data Availability

The raw data of the current study are available from the corresponding author on reasonable request.

## Abbreviations

PONV: Postoperative nausea and vomiting
US: Ultrasound
TAPB: Transversus abdominis plane block
RSB: Rectus sheath block
ASA: American Society of Anesthesiologists
PCA: Patient-controlled analgesia
PCIA: Patient controlled intravenous analgesia
IV: Intravenous
BIS: Bispectral index
TEM: Total mesorectal resection
CONSORT: Consolidated Standards of Reporting Trials
PACU: Post-anesthesia care unit
NRS: Numerical rating scale
APS: Acute pain service
FAS: Functional activity score
